# Policy implementation: Assessing institutional coordination and communication for flood warning in Namibia

**DOI:** 10.4102/jamba.v16i1.1534

**Published:** 2024-07-31

**Authors:** Deolfa R. Jose Moises, Nnenesi A. Kgabi, Olivia Kunguma

**Affiliations:** 1 Disaster Management Training and Education Center for Africa, Faculty of Natural and Environmental Sciences, University of the Free State, Bloemfontein, South Africa; 2 Department of Civil and Environmental Engineering, Faculty of Engineering, Namibia University of Science and Technology, Windhoek, Namibia; 3 Department of Biochemistry, Microbiology and Biotechnology, Faculty of Agriculture, Engineering and Natural Science, University of Namibia, Windhoek, Namibia; 4 Unit for Environmental Sciences and Management, Faculty of Natural and Environmental Sciences, North-West University, Potchefstroom, South Africa

**Keywords:** DRR streamlining, governance, institutions, coordination, policy implementation, early warning systems

## Abstract

An effective institutional coordination and communication are essential determinants of community disaster resilience capacities and successful execution of disaster risk reduction (DRR) strategies. Several international agreements endorse adopting consolidated, decentralised, participatory approaches to manage hydrometeorological hazards. Yet, the capacity of local governments to develop and implement effective disaster risk reduction strategies remains inconsistent and relatively unknown. In its quest to achieve sustainable economic development, Namibia has developed a DRR legislative framework and enlisted a central body to execute the strategic policy for disaster disruption mitigation and national resilience building. This study adopts a qualitative case study approach to assess the role of the central disaster risk management institution in establishing effective institutional coordination and communication structures for successful flood early warning system operations. Based on its legislative mandate, the study presents a hierarchically contextualised account of the institutions’ empirical progress in DRR streamlining.

**Contribution:**

The study identifies bureaucracy, limited institutional capacities, inadequate funding and response and relief prioritisation as major challenges to system efficacy. It provides directives for better institutional coordination and communication to reduce future harm.

## Introduction

As a system continuously subjected to the proliferation of multiple key players, the evolution of global disaster risk reduction in the last 20 years has been monumental. Disaster risk reduction (DRR) has become a collective global initiative in which all parties have consented to their roles, which has seen the international community augment directives from single response-driven efforts to more diversified mandates almost annually (Lassa 2018). However, ensuring the proactive manifestation of DRR practices from national to local levels that benefit the most vulnerable communities is still a pressing matter (Gaillard & Mercer 2013).

In March 2015, the United Nations World Conference on Disaster Risk Reduction gave way to adopting the Sendai Framework for Disaster Risk Reduction (SFDRR) by 187 member states, which outlines a set of action priorities for DRR. Under priority two, the nations set out to firstly ‘establish a designated national focal point for implementing the SFDRR’ and secondly ‘ensure that disaster risk reduction is a national and local priority with a strong institutional basis for implementation’ with ‘clearly assigned responsibilities and authority that facilitate and support local multisectoral cooperation (e.g. among local governments) for disaster risk reduction’ based on ‘laws, regulations, standards’ (United Nations Office for Disaster Risk Reduction 2015). Since then, significant enhancements to and adoption of relevant legislation have ensued; however, gaps remain, particularly with implementation at community levels (UNDRR 2023).

The mid-term review of the SFDRR reveals that although most member states have successfully established regional and national focal institutions and DRR plans, progress for local hazard risk governance and DRR decentralisation approaches have been delayed (UNDRR 2023). Similarly, multidisciplinary and integrated coordination strategies have been limited. Centralised policies and institutions seemingly inhibit the adoption of DRR policies into developmental plans. Several participants for the review indicated restricted mandates in policy implementation at local levels, even where DRR legislation is available (UNDRR 2023).

Furthermore, the aftermath of high-impact disasters such as hurricanes Maria and Katrina in North America saw several researchers emphasise the need to capacitate local institutions and limit bureaucracy to ensure effective DRR response from the grassroots level (Farber 2018; Tselios & Tompkins 2017; Wilkinson 2012). A study on the role of local governments in DRR by David (2015) asserts that streamlining DRR from national to local institutions proves effective as these institutions are vital to the division of roles and responsibilities, identification and support of vulnerable groups, establishment of effective communication networks and emergency decision-making. Because local communities and institutions are the first participants to disaster, it is no surprise that the international community acknowledges and supports preparedness and response efforts at this level (Cowan, O’Brien & Rakotomalala-Rakotondrandria 2014; IFRC 2014). Many studies have emphasised the need to ensure that those tasked with DRR streamlining form effective networks for sustainable implementation at all governmental levels (Das & Luthfi 2017; Scott & Tarazona 2011). Due to the apparent incremental costs between preventative versus response and relief initiatives (Shreve & Kelman 2014), developing countries under-prioritise the former (Wilhite 2002), leaving extensive skills and capacity gaps for DRR operations at subsequent governmental levels (Messer 2003).

This study assesses the legislatively mandated role of the focal DRR institution, the Directorate for Disaster Risk Management (DDRM), in establishing effective institutional coordination and communication networks for early flood warning in Namibia. Given the study’s limited scope, it does not attempt to dissect all legal and institutional frameworks relevant to DRR in Namibia comprehensively. Rather, it presents a contextualised overview of DRR streamlining and its effect on flood risk reduction by contrasting the legislative protocols outlined in the *Disaster Management Act* with practice.

### Flood early warning communication and response coordination in Namibia

Namibia is vulnerable to various anthropogenic and natural hazards, especially flooding (Reid et al. 2008). Floods are an annual recurrence, worsening every year and gravely impacting the northern and northeastern regions of the country (UNDRR 2019). In the past, flood-fighting initiatives were almost solely centred on relief efforts. The colonially inherited *South African Civil Defence Act* and Ordinances, or the *Civil Defence Act 1966* as it was known in Namibia, governed all disaster management matters until the promulgation of the *Disaster Risk Management Acts* (DRMA) in 2012 (GRN 2013; Republic of South Africa 1966, 1969).

The Act postdated the undeniable need for policy-driven hazard risk reduction strategies following the devastating impacts of the 2009–2011 flood disasters in the country (Hosseini-Boroujeni 2019) and is supported by two other pieces of legislation, the National Disaster Risk Management Plan (2011) and the National Disaster Risk Management Policy (2009) (GRN 2011, 2013). The development of this framework (comprising these three pieces of legislation) presented a step towards the global paradigm shift from disaster response to an integrated multi-hazard strategy focusing on hazard risk reduction (Van Niekerk 2008). Aligning well with several relevant international agreements, that is, the SFDRR, the African Regional Strategy for Disaster Risk Reduction (ARSDRR) and the Kyoto Protocol, the framework outlines a well-structured approach to DRR (African Union 2004; UNDRR 2015; United Nations 1998), even highlighting the relevance of and promising the immediate establishment of early warning systems (GRN 2013). However, several reports suggest that this is yet to manifest empirically, as several communities have continued to bear the seemingly unmitigated brunt of flood events (Mabuku et al. 2018; Mashebe 2015; Shaamhula, Smit & Van der Merwe 2021).

The main objective of the Flood Early Warning System (FEWS) in Namibia is to provide at-risk communities with information and alerts of impending hazards and directives for evacuation (Lumbroso 2018). To this end, several organisations operating in the managerial and technical facets of disaster preparedness collaborate to provide civil society with flood information (Mandl & Frye 2012). These systems require an accurate and timely forecasting system, robust and continuous communication network, reliability and collaborative action to meet this objective (Cools, Innocenti & O’Brien 2016). However, floods have persisted in frequency and impact, with failed evacuations and losses often credited to poor communication and coordination (National Planning Commission 2019).

In the assessment for African floods in 2021, the International Federation of Red Cross and Red Crescent Societies lists Namibia as the country with the highest population ratio exposed to flood risk (IFRC 2021). Approximately $100 million of GDP, corresponding to about 0.9% of the total GDP per annum, is at risk of direct loss by flood impact (UNDRR 2019). Apart from experiencing major flooding from 2008 to 2011, in which over $136m in damages and $78m in direct economic loss were sustained in 2009, Namibia is exposed to high-impact flooding annually, impacting over 54% of the total population (Government of the Republic of Namibia 2009).

The seemingly unmitigated flood recurrence has raised questions about why DRR has yet to receive due attention at all governmental levels in Namibia. Why do developed policy-based institutional and communication networks not deliver on the ground? What are the policies, structures and institutions that hinder effective DRR in the country? To help address these concerns, this paper systematically assesses the roles, responsibilities, and success of the focal disaster management institution, the DDRM, in streamlining DRR across all governmental levels and how this has translated in reference to flood early warning in Namibia.

## Research methods and design

The primary purpose of the Namibian *Disaster Risk Management Act* is to facilitate the administration and coordination to execute its four main objectives:

1. ‘To ensure provisions and establishment of DRR institutions in Namibia
2. ‘to ensure and maintain provisions for a multisectoral coordinated DRM strategy that prioritises risk prevention and reduction, preparedness, response and recovery’
3. ‘to provide for declaration of disasters’
4. ‘to establish the Disaster Risk Management Fund’ (GRN 2013).

**FIGURE 1:**
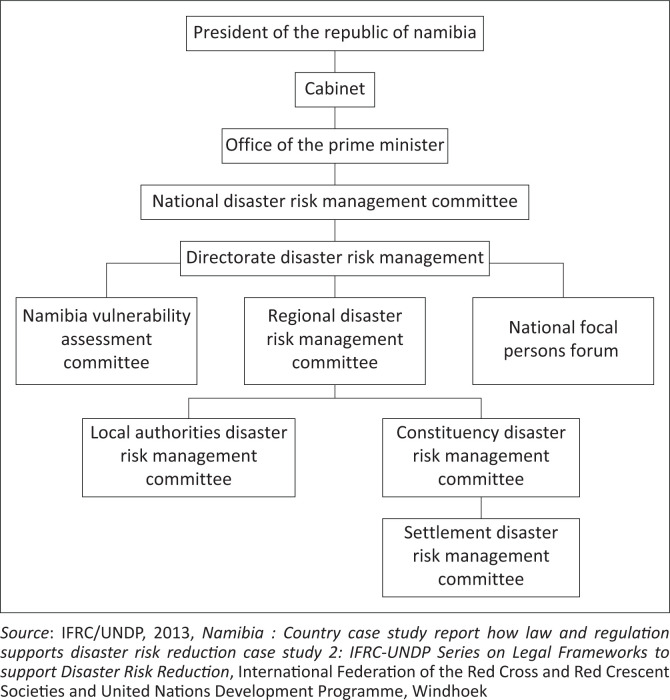
Institutional framework for Disaster Risk Management in Namibia.

At the highest level, current institutional responsibility for DRR in Namibia rests with the Office of the Prime Minister (OPM), which executes decisions formulated by the National Disaster Risk Management Committee (National DRMC) via the DDRM which is the institution responsible for the day-to-day administration of DRR matters in the country (Figure 1) (GRN 2013; IFRC/UNDP 2013).

According to the DDRM’s tasks outlined in the *Disaster Risk Management Act* (Section 11.4), the institution is responsible for establishing and maintaining regional, local authority and settlement Disaster Risk Management Committees (DRMCs) to coordinate and execute DRR activities at these subsequent administrative levels (GRN 2013). Concerning FEWS, the DDRM’s responsibilities further include the following:

- ‘To facilitate the development, strengthening and transformation of the disaster institutions’
- ‘facilitate and coordinate disaster risk assessments undertaken in partnership with stakeholders, regional councils and local authorities’
- ‘facilitate and coordinate the development, implementation and maintenance of specific disaster risk reduction strategies, aimed at building resilient areas, communities, households and individuals’
- ‘the development and testing of contingency plans of known priority risk at all levels of government’
- ‘the development of response and recovery plans to ensure rapid and effective response to disasters that are occurring or are threatening to occur and to mitigate the effects of those disasters that could not have been prevented or predicted and’
- ‘to align and consolidate national early warning systems’.

According to Chisty et al. (2022), assessing progressive legislation and policies is essential for effective DRR, as these efforts are often marred by poor implementation.

### Data collection and analysis

**FIGURE 2:**
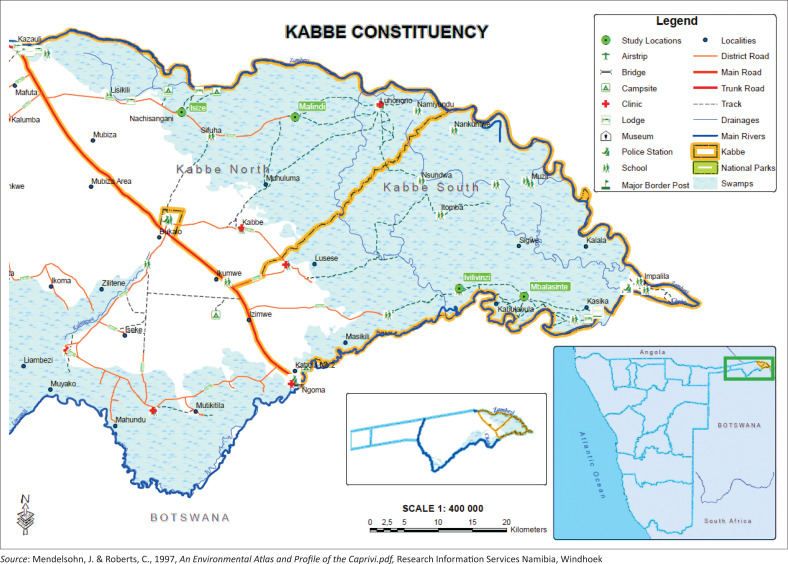
Kabbe constituency.

The study adopted a case study approach, which included the review of literature, the legislative framework (Figure 1) and supporting policies, supplemented with qualitative data obtained through purposive key informant interviews (KIIs) and focus group in the Kabbe constituency (Figure 2). Characterised by annual riverine and flash floods and currently categorised as the area most sensitive to flood impact in Namibia, the researches deemed it most appropriate for the assessment. The researchers assessed the DDRM’s establishment of effective institutional coordination and communication networks for flood early warning in Namibia by collecting data on the extent of DRR streamlining through (1) an overview of the technical operationalisation of the FEWS through KIIs with DRR officials and (2) the experiences and perspectives on flood risk and reduction of impacted communities through FGDs. The information was then compared against the strategy outlined in the DRMA. The combination of data collection tools also allowed for cross-validation of information, increasing the study’s reliability.

Acknowledging the composite nature of flood disasters, the selection of nine KII participants comprised a diversity of national to local level government DRR officials, the Namibia Red Cross, which actively collaborates with communities to manage flood risk and community leaders (i.e. village chiefs and church leaders) directly involved in early warning systems operations. The interviewees were selected based on their expertise, experience and involvement at particular levels within the flood early warning process.

The interviews were based on semi-structured questions structured around the DRM Act 10 of 2012 which outlines DDRM’s duties. Some of the questions included: What is the role of the DDRM in establishing stakeholder relations and communication flow among early warning system institutions at all governmental levels? Has the DDRM established DRR institutions for early warning coordination and information management at all levels of government? How does the DDRM ensure community engagement in early warning systems processes across all governmental levels? What is the extent of the DDRM’s implementation of its roles and responsibilities outlined in the DRMA and support policy concerning the flood early warning system?

Assisting communities in successfully combating flood risk was the impetus for assessing the DDRM’s implementation of the DRMA. As such, the study saw it necessary to draw on communities’ perceptions, experiences and opinions to ascertain good practices and challenges and establish how society and its concerns are integrated into the institutional framework. To conduct this analysis, eight FGDs were conducted with residents from the four most flood-affected communities in Kabbe, located both up and downstream in the study area and homes of two different ethnic groups, which represented possible disparity in, for example, risk perceptions, preparedness methods, influence of customs of dealing with floods, among others. A total of 59 participants were selected based on:

- Their age (> 35 years).
- Experience of the 2009 and 2011 Namibia flood state of emergency and latter flood occurrence in the area.
- They had to be the head of a household.

The selected participants included members from community-driven organisations such as the Red Cross-established Community Disaster Risk Management Committees (CDRMCs), members of community organisations and regular residents. The diversity within the selected sample provided additional information on the extent of DRR decentralisation by enabling the assessment of the level of community involvement in the planning, communication and response of the early warning process. Some questions asked included: Are residents aware of the national flood early warning system? Are residents aware of what governmental institutions to contact during a flood? How often are the residents engaged in government-led flood DRR initiatives? What are the main sources of flood information in your locality?

The area was selected for two reasons:

1. Its recurrent exposure to flooding, which is representative of a rich body of knowledge and experience in dealing with flood disasters.
2. The presence of the Namibia Red Cross in the area over a decade after the last national state of emergency in 2011, which was indicative of possible unknown factors influencing the seemingly unmitigated flood impact in the area, that is, dependency syndrome. The information from both the KIIs and FGDs were thematically analysed as a single data set and presented in themes outlined in section ‘Results’. Whereas the KIIs provided an overview of the institutional communication and coordination structures for flood early warning through DRR streamlining, the FGDs served to corroborate and support or debunk the said information by providing insight into the affected communities’ actual involvement or lack thereof within this framework.

### Ethical considerations

Ethical clearance was granted by the General/Human Research Committee at the University of the Free State under Research Project Title: Analysis of the Operational Flood Early Warning System in Kabbe Constituency, Zambezi Region – Namibia. Ethical clearance number: UFS-HSD2022/0400/22.

## Results

The nature of governance, hierarchies and other relevant structures influence communication, decision-making, and coordination within an early warning system. Similarly, their internal governing and hierarchical structures define key stakeholder institutions mandated to operate these systems, with overall efficiency often determined by their distinct financial and human resources capacities. Consequently, the overall governing structures, institutional capacities and political dynamics also affect vulnerable communities as they all lead to a common thread of decisions and actions during a flood emergency.

Considering the complexity of these relationships, the study contrasts policy requirements and empirical data from KIIs and FGDs to assess the DDRM’s role in establishing and maintaining effective institutional and communication structures for impact-based flood early warning. The results are presented in selected themes (presented below) and illustrate how overall FEWS performance and efficacy are governed by a multiplex of relationships influencing overall systems communication and coordination.

### Coordination and information management

The study confirmed that at the national level, the DDRM is the country’s custodian for all DRM affairs and is considered the government’s disaster management agency. Assuming its primary function as stakeholder coordination, participants at the national level agree that, at this level, coordination is somewhat effective. However, interviewees suggested the establishment of an effective information management and communication system and a better definition of roles and mandates to strengthen coordination and communication among different agencies. Although not legally mandated, the DDRM is responsible for several sectoral units that feed into the NDRMC flood early warning decision-making process (i.e. NGOs, I/NGOs, private sector). Although these agencies maintain contact during DDRM-led meetings, communication linkages among themselves are often limited:

‘We have our quarterly meetings as stipulated in the act. However, there is almost never any follow-through or communication between the relevant parties within those intervals. Furthermore, information sharing is minimal. We source data and information from where we can, e.g. the agencies we work with, such as Meteorological Services of Namibia and NamWater. However, information or data directly from institutions we don’t usually engage with proves difficult or costly.’ (Ministerial Official, Interviewee OPM01, 14 JUL 2022)

Furthermore, data sharing among agencies is minimal. However, some institutions produce information, for example, risk maps, that can be used in flood forecasting and monitoring, but these agencies are not formally involved in the FEWS process. In addition, the FGDs coroborate these findings by pointing out the lack of government-led risk awareness and communication efforts supplemented by the observable unavailability of risk maps in regional and local level offices and communities. On the contrary, interviewees agree that existing formal communication networks work relatively well at the national level. However, the same cannot be said for subsequent governmental levels.

### Regional level disaster risk reduction implementation

The study established that a Regional DRMC (RDRMC), tasked with all DRR efforts, including FEWs initiatives, has been replicated and is fully functional. The KIIs with officials from the Zambezi Regional Council, Local Authority and the Namibia Red Cross from field visits to the Kabbe confirmed that the Regional DRMC was responsible for FEWS activities in Kabbe and that the Regional Disaster Management Committee (RDMC) met occasionally. The KIIs further revealed that the personal capacity of the RDMC was limited to a single permanent staff member, with no designated department and limited resources available for flood early warning activities, including emergency response.

The participants also revealed the ongoing response-driven nature towards flood risk management by the DDRM and inadequate departmental awareness of DRR awareness and long-term preparedness and resilience. This can be attributed to inadequate resources, planning and training and the need to prioritise time and available resources for response rather than preparedness and mitigation:

‘I am the only designated DRR staff in the regional council, and as you can see, we have no department. I believe that DRR functions would be executed more successfully if we had more manpower and if officials understood DRR. Nobody wants to take responsibility; everyone believes that we are just assisting and this is a job for the national government.’ (Zambezi Regional Council official, RDRMC Member, Interviewee ZRC01)

A major criticism levied by sub-national participants is that the RDRMC comprises Zambezi Regional Council staff primarily and the committee often meets on an ad hoc basis when flood disasters or emergencies occur. As such, ensuring DRR implementation and ownership at these governmental levels is often difficult as very little time is assigned to planning and effecting long-term strategies. Participants further revealed recurring absenteeism and substitutions by unversed proxies at RDRMC meetings, demonstrating that not all committee members prioritise their duties. This is partly attributed to members’ perception of the meetings as an added burden to their ‘regular workload’.

However, good practice was observed in the open and functioning communication lines between the DDRM and RDRMC. The regional council official also praised the DDRM’s involvement in regional DRR matters. On the contrary, a major contingency plan that was the development of the Regional Emergency Operations Centre (REOC) for which preparations began in 2013, is yet to be established. Furthermore, communication networks among regional institutions are non-existent and emergency response coordination often takes the form of personal favours among officials from different institutions or assistance is requested from the DDRM:

‘We don’t have the resources to conduct emergency response independently. We usually request help from other ministries in the region, and they help where they can, but if they can’t, we have no choice but to contact the DDRM. Right now, our evacuation boat is damaged, and we wouldn’t know how to transport people if a flood were to hit.’ (ZRC Official, DRMC Member, Interviewee ZRC01)

### Disaster risk reduction decentralisation and decision-making capabilities

Another challenge brought forward at the regional level is poor DRR decentralisation and the red tape that delays FEWS’ functions at the regional level. The DDRM approves sectoral budgets, which are often delayed and contingent upon what issues the government considers priorities. Furthermore, although the regional office is extended the mandate to plan flood DRR initiatives, budgetary constraints limit their scope of implementation to emergency response. Although the DDRM is supposed to ensure that the RDRMCs coordinate and implement flood preparedness, mitigation, response and recovery efforts, the Namibia Red Cross independently steers these efforts:

‘We conduct monitoring and evacuation training with community DRMCs and other groups. The Regional Council and Local Authority are not really involved. However, we do inform them of our progress and exchange information during the flood season.’ (Namibia Red Cross official, Interviewee NRC01, 19 JUL 2022)

### Local and community-level implementation

The KIIs and FGDs revealed significant gaps in local and community-level DRR prioritisation and implementation. Firstly, constituency and settlement DRMCs have yet to be established under the guidelines provided by the DRMA; secondly, DRR functions are the designation of a single official at the constituency level with no personnel at the settlement level, an indication of limited capacity. It is expected that NGOs will compensate for capacity building in this area. Similarly, government-established community DRMCs and local authority volunteers are non-existent. Although communities acknowledge receiving flood warnings and relief from authorities at times, communities are unaware of the existence of a national FEWS. This can be attributed to the lack of DRR ownership at the local administrative level:

‘We often see government officials come around and warn us of impending floods, and they provide relief depending on how bad the flood is. However, we were not aware of the early warning system. The Namibia Red Cross is the only institution that conducts training for preparedness, mitigation, response and recovery as far as we know.’ (Resident in Malindi, Interviewee M09, 20 JUL 2022)

Although, in theory, communities are regarded as the most relevant stakeholders in the FEWS process, the FGDs and KIIs reveal that, in practice, this is not the case. Residents and government officials confirm that communities are only engaged within the FEWS chain during the alert and evacuation process. Regional officials attribute this to a lack of funding for conducting any type of outreach but argue that the Red Cross conducts several important functions, for example, risk mapping, evacuation planning, identification of vulnerable areas, among others.

Moreover, the FGDs highlighted poor knowledge of DRR legislation and practice among most residents and a lack of opportunities for community engagement. While acknowledging the sense of ownership provided by the Red Cross’s initiatives, residents simultaneously expressed feeling left out and disconnected from decision-making by national and sub-national government institutions. However, it is imperative that these grievances are contextualised within the general scope of community collaboration in developmental matters in Namibia.

Firstly, participatory natural resource management (involving communities) is an effective and relatively common practice in Namibia and several NGOs support and manage community-based developmental programmes (Naidoo et al. 2011). Secondly, many participants belonged to community organisations, committees and volunteer groups and contrasted their decision-making power within these organisation against their experiences or lack thereof with the government:

‘The Namibia Red Cross only consults us on matters with the floods. They want to know our history, coping methods and how they can assist.’ (CDRMC Member, Interviewee I13, 20 JUL 2022)

Conversely, regional and local authority officials observed bureaucracy, inadequate financial and human resources and slow-paced decision-making as challenges for inducing community participation. Whereas national-level officials promise a change in this direction, regional and local authorities expressed frustration at the inability to execute their own decisions. In addition, the available scope for participatory decision-making is limited to necessity and traditional authorities who may or may not consult their residents.

A key-note feature of the study was the prevalence of female FGD attendees. Although it can be argued that men in rural communities often travel for work, the prevalence of women in the village committees proves that their needs are prioritised. Female participants expressed feeling well-represented in community organisations and were included in decision-making. The KII, with an NGO, referred to women as primary contact points and beneficiaries of flood warnings in rural areas. Finally, the KIIs revealed that the consolidation of national early warning systems was still in the planning stages, explaining some gaps identified in multisectoral communication.

## Discussion

Disaster risk reduction policies and strategies can be more effectively implemented if effective institutional coordination and communication are established at all levels of government. This requires a collective understanding of risks, community needs and available capacities at all governmental levels, including inter- and multi-sectoral relationships among all relevant stakeholders. Along with sufficient financial investment, effective DRR requires establishing participatory planning and implementation approaches, as all stakeholders have a significant role in mitigating, preparing for, responding to and recovering from the impacts of disasters.

In the light of Namibia’s flood vulnerability profile, this study assessed the DDRM’s role in establishing institutional coordination and communication structures for effective flood early warning processes. Bearing in mind the novelty of the institution, the legislation governing its mandate, and the financial deprioritisation of DRR, it may take more time than expected to successfully implement effective disaster management strategies at sub-national levels. The analysis of the DDRM’s policy implementation emphasised the lack of strategic focus on capacity building, mitigation and preparedness through the revelation of the ongoing prioritisation of short-term response and relief approaches. In terms of alleviating vulnerabilities and improving livelihoods, legislative, institutional and regulatory shortcomings have remained a hindrance (Ferrol-Schulte et al. 2015).

As a result of the bureaucratic nature of DRR governance, communities have become more self-reliant in addressing flood risk, participating in several NGO and local flood DRR initiatives. However, poor community engagement has hindered the government’s ability to exploit these efforts. This is largely attributed to the lack of an accountability system, disregard for the lessons learnt from flood disasters that formulated the basis for policy development, poor definition of roles, institutional inertia and poor DRR decentralisation.

Furthermore, the DDRM has failed to expand institutional coordination and communication structures to sub-national levels, with several regional, constituency and settlement institutions yet to be established as far as 11 years into the promulgation of the DRM Act and other support legislation. Moreover, the existing sub-national DRR institutions have not received the financial support required to develop, implement and maintain DRR needs at these levels. Although local governments play a critical role as primary administrative participants to flood risk, the DDRM has failed to leverage their strategic position as conduits to communities to establish local structures to access external resources for risk reduction and coordinate and support communities in the early warning process. According to Wilkinson (2012), institutional proximity to vulnerable groups is integral to implementing DRR strategies, especially at the beginning stages.

Another significant challenge highlighted by the study was the disregard for civil society organisations in DRR planning. Most of these organisations are able to penetrate the most remote and vulnerable areas that are often inaccessible to governmental institutions and directly assist communities with preparedness, response, relief and recovery efforts. The need to include these actors in the institutional DRR structure ensures effective coordination and impact-based results across all phases of the FEWS cycle. The inclusion of these communal, religious and civil actors requires incorporation into national policies and DRR streamlining in developing countries (Gero, Méheux & Dominey-Howes 2010). To attain a level of effective coordination and communication to improve the FEWS in Namibia, the DDRM needs to investigate and incorporate integrative-holistic DRR governance strategies with less bureaucracy, develop mechanisms for including local experiences, knowledge and expertise and establish and strengthen institutional capacities for DRR, mitigation, preparedness and management.

Based on the identified gaps, recommendations to improve both policy and implementation were provided. These are summarised in Table 1.

**TABLE 1:** A summary of findings, gaps and recommendations.

Identified gaps and findings	Recommendations
DDRM issues warnings and evacuation orders Centralised system DDRM is the regulator and validates warning. However, the system is operated and warnings are generated by the MoA Overarching authority of the DDRM Clear hierarchy at the national level DDRM determines the funds to be assigned towards DRR at all governmental levels. Based on approval	Develop decision-making mandates at sub-national levels to aid in DRR decentralisation. Allow sub-national budget development and approvals. Analyse the existing national through community-level DRR plans, operations and protocols for hazard emergencies to strengthen policies. Evaluate mandates/roles of the DDRM and other national to community DRR agencies/organisations, identify gaps and address them using relevant multidisciplinary and research-based strategies. Identify and develop partnerships between all relevant stakeholders. Develop a working platform to unite DRR specialists and early warning end-users to promote better understanding of the distinct and evolving needs of end-users, especially the last mile, to jointly identify, design, and implement strategies and actions to improve risk and warning communication and response coordination. This platform (which could include stakeholder workshops) will address content, format, delivery, lead time, communication of technical limitations (including uncertainty), literacy and gender.
Different practices at the local level No decision-making authority at regional and local levels Absence of hierarchy between regional and local authorities	Develop SOPs at regional and local levels. Define the mandates, roles and jurisdictions of each institution.
Both MoA and Regional authorities are involved in warning dissemination and maintain contact with external service providers	Define the roles of each institution. Minimise discrepancies in practices among same-level institutions.
Discrepancies in SOPs for local circumstances, that is, undefined roles and communication frameworks at regional and local levels	Establish SOPs within regional and local authority institutions and levels. Develop technical and funding plans to enhance DRR streamlining, communication infrastructure and networks, especially at sub-national levels.
Absence of integrated SOPs for all stakeholders	Establish SOPs at each governmental level, with all relevant institutions for overall FEWS.
Absence of government-established DRMCs at local authority and community levels A lack of coordination between stakeholders at regional and local levels	Increase capacity and tools for coordination. Provide SOPs on coordination and communication. Develop DRMCs at local and community levels.
Institutional roles switch according to circumstances Inadequate capacity and training at the regional and local level	Clearly define the roles of individual institutions and officials within them, especially at regional and local levels. Develop standardised mandate to appoint and retain trained and specialised staff. Increase funding to human resource development.
A lack of risk awareness campaigns and communication structure at regional, local and community levels	Improve FEWS through research and development. Establish alternative means of communication in the failure of the standard warning chain. Establish clear mechanism to receive feedback before, during and after flood emergencies. Increase community participation through awareness campaigns, response and evacuation drills.
Many actors in the FEWS process (at all levels) are not experts in the field	Disburse funding towards skills and knowledge training to improve capacities.
A lack of community engagement across all levels A lack of community engagement in planning and response, which demonstrates a poor understanding of their needs The Red Cross liaises with communities, and all CDRMCs were founded based on this relationship	Establish pathways for community engagement across all levels by establishing committees that table public concerns for consideration at all governmental levels with a feedback loop. Collect data on FEWS efficacy at all levels. Develop appropriate products and data and information packages for end-user to create risk and preparedness awareness. This information would include emergency contacts and how and when communities should go about communicating hazard information. This would assist in ensuring effective communication and coordination structures at levels proportionate to the magnitude of the flood. Develop training and capacity-building initiatives for early warning end-users to improve user product development, delivery, usability, evaluation and interpretation.

DDRM, Directorate for Disaster Risk Management; MoA, Ministry of Agriculture; DRR, disaster risk reduction; SOP, Standard Operating Procedures; FEWS, Flood Early Warning System; CDRMC, Community Disaster Management Committee.

### Limitations and delimitations

This study presented several limitations:

- The study focused on DRR streamlining based on a single hazard.
- The study focused on the focal DRR institution’s, that is, the DDRM’s, role in establishing a comprehensive coordination and communication structure for effective flood early warning.
- The study was based on the institutional roles outlined in the DRMA and support legislation, that is, the National DRM Plan and the National DRM Policy.
- Time constraints and the unavailability of certain key individuals to participate limited the number of participants that could be interviewed.

These limitations were delimited by the fact that:

- Flood risk is the most impactful national hazard and a national priority area.
- The DRMA is a fairly new piece of legislation, and DRR decentralisation began post-2011 with minimal reporting on the DDRM’s progress.
- The review of literature and pre-interview consultations with officials at the DDRM and subsequent DRR institutions confirmed that the reviewed legislation was most relevant for assessing the flood early warning system.
- The researchers selected key individuals in every target institution and requested their recommended seconder where they were unavailable.

## Conclusion

Over the past two decades, the escalation in the magnitude and frequency of hydrometeorological hazards such as floods has resulted in the need to develop robust and effective strategies to minimise the loss of lives and damage brought on by these events. Leading this global effort is the development and implementation of early warning systems, which require real-time data sharing and multilevel coordination from governmental institutions for optimum efficacy. Based on the country’s administrative boundaries, this often includes the integrated collaboration of national, regional, provincial, municipal, local levels, non-governmental organisations, the private sector, agencies in charge of meteorological and climate services, and the primary authority for DRR regulation. The institution responsible for DRR regulation is of primary significance and is often responsible for DRR streamlining, which establishes overall warning communication and response coordination activities. In developing and least-developed countries, the establishment of keynote DRR institutions is often lacking, leaving the task to the national hydrometeorological service provider. Whereas where they exist, the capacity to effectively streamline DRR to all administrative levels is often limited. In most cases, the interfaces and linkages between these agencies and other institutions are often weak or non-existent, posing challenges in effectively developing and implementing these systems. Hence, an urgent need to assess, strengthen, and formalise collaborations while addressing hydrometeorological hazard risk reduction.

Based on the key findings presented in this study, it is evident that significant developments and enhancements to DRR policy and practice are required to address the myriad of gaps contributing to poor communication and response coordination within the FEWS process in Namibia. However, the availability of guiding policies, existing institutions, and operating structures geared towards flood DRR demonstrate a willingness to tackle hazard risk head-on. Moreover, adding to the existing gaps identified across different governmental levels, several ‘challenges’, such as the prioritising of other developmental needs, poor DRR financing, among others, influence the DDRM approach to DRR streamlining and, thus, the overall communication and response coordination within the flood warning process. These challenges cannot be addressed by mere institutional reforms but require concerted efforts by the government, at policy development and developmental planning levels, to ensure that DRR planning and streamlining approaches align with developmental plans and are implemented in an integrated and cohesive manner.

As a concept that encourages early intervention to minimise hazard impacts, flood early warning systems are a complex DRR strategy that bears unique challenges and often requires several interlinked and robust yet flexible structures for optimum output. Early intervention strategies, by their nature, require effective pre-planning (i.e. the establishment of well-tested communication and response coordination networks) and immediate decision-making and are defined by their ability to generate immediate results. As such, in its approach to successfully streamline DRR, particularly for flood early warning, the DDRM will have to take into account the unique circumstances (social, environmental and economic) that define and govern these systems and all additional factors that influence DRR planning and implementation such as developmental plans to improve flood DRR in the country. The study recommendations for policy and practice provided in this article serve as a stepping stone towards the journey to strengthen flood risk and warning communication and response coordination and overall flood DRR in the country.
